# Biological Effects of Culture Substrates on Human Pluripotent Stem Cells

**DOI:** 10.1155/2016/5380560

**Published:** 2016-08-30

**Authors:** Yohei Hayashi, Miho Kusuda Furue

**Affiliations:** ^1^Laboratory of Gene Regulation, Faculty of Medicine, University of Tsukuba, 1-1-1 Tennodai, Tsukuba, Ibaraki 305-8575, Japan; ^2^Laboratory of Stem Cell Cultures, National Institutes of Biomedical Innovation, Health and Nutrition, 7-6-8 Saito-Asagi, Ibaraki, Osaka 567-0085, Japan

## Abstract

In recent years, as human pluripotent stem cells (hPSCs) have been commonly cultured in feeder-free conditions, a number of cell culture substrates have been applied or developed. However, the functional roles of these substrates in maintaining hPSC self-renewal remain unclear. Here in this review, we summarize the types of these substrates and their effect on maintaining hPSC self-renewal. Endogenous extracellular matrix (ECM) protein expression has been shown to be crucial in maintaining hPSC self-renewal. These ECM molecules interact with integrin cell-surface receptors and transmit their cellular signaling. We discuss the possible effect of integrin-mediated signaling pathways on maintaining hPSC self-renewal. Activation of integrin-linked kinase (ILK), which transmits ECM-integrin signaling to AKT (also known as protein kinase B), has been shown to be critical in maintaining hPSC self-renewal. Also, since naïve pluripotency has been widely recognized as an alternative pluripotent state of hPSCs, we discuss the possible effects of culture substrates and integrin signaling on naïve hPSCs based on the studies of mouse embryonic stem cells. Understanding the role of culture substrates in hPSC self-renewal and differentiation enables us to control hPSC behavior precisely and to establish scalable or microfabricated culture technologies for regenerative medicine and drug development.

## 1. Introduction

Human pluripotent stem cells (hPSCs) have the unique features of self-renewal and pluripotency. These features give rise to the unprecedented potential of advancing regenerative medicine, drug development, and human biology. Two types of hPSCs are widely used; human embryonic stem cells (hESCs) were derived from the inner cell mass (ICM) of the blastocyst [1], and human induced pluripotent stem cells (hiPSCs) were generated by introducing key transcription factors into somatic cells [2, 3]. There have been numerous arguments for differences between hESCs and hiPSCs; however, both hESCs and successfully-reprogrammed hiPSCs generally have similar gene expression patterns, differentiation potentials, and epigenetic signatures [4–6].

To utilize hPSCs in industrialized regenerative medicine and drug development, the culture methods must be improved to meet the technological standards in safety, cost-effectiveness, and the easiness of handling. Early culture methods for hPSCs were similar to the methods developed for mouse embryonic stem cells (mESCs) [7, 8]. The methods employed coculturing hPSCs with irradiated or mytomicin-C-treated mouse embryonic fibroblasts (MEF) or immortalized embryonic fibroblast lines (e.g., STO or SNL cell lines) as feeder cells in culture media containing fetal calf serum (FCS) or serum replacement (SR). Since these feeder cells, FCS, or SR provide undefined attachment factors for cultured cells, specific culture substrates except for gelatin, which are hydrolyzed and denatured collagens, are not generally required. Since feeder cells, FCS, or SR also contain undefined, xenogenic allergens, these components must be carefully audited for animal-derived raw material controls in compliance with regulation in applying to regenerative medicine. Besides, using feeder cells practically caused many troubles, such as cellular cross-contamination, the lack of reproducibility, or time-consuming preparation.

To overcome these problems, feeder-free culture methods have been developed. The first study used the conditioned medium of MEF feeder cells in 2001 [9]. In 2004-2005, subsequent studies used the high concentration of SR or other crude extracts supplemented with recombinant cytokines and/or chemicals [10–13]. Since 2005, defined culture media for hPSCs have been developed [14–18]. In these culture media, the most common cytokine is bFGF, which activates MAPK and/or PI3K-AKT signaling pathways. The second is Nodal/Activin/TGF*β*, which activate TGF*β*-SMAD signaling pathway. Activation of these signaling pathways is critical to maintain hPSC self-renewal [15, 19]. Defined culture media enable us to identify other cytokines and chemicals which promote hPSC self-renewal and/or single-cell survival (e.g., heparin [20], albumin-associated lipids [21], pleiotrophin [22], and IGF1 analog and heregulin-1*β* (a ligand for ERBB2/ERBB3) [23]). Extracellular Matrix (ECM) proteins and other culture substrates can be also effectively examined using defined culture media.

Here we summarize the application of various ECM proteins and other types of culture substrates to defined hPSC culture media. Further, we will discuss the functional roles of signaling pathways under integrins, which are major receptors for ECM proteins. Last, we will discuss the possible effect of culture substrates on naïve hPSCs, which exhibit the different biological state of pluripotency; although conventional (or called “primed”) embryonic or induced pluripotent stem cells cannot create chimeric animals by the blastocyct injection, naïve pluripotent stem cells can create them. Naïve and primed pluripotent stem cells have distinct gene expression patterns and signal dependencies [24].

## 2. Role of Culture Substrates in hPSCs

Although some studies have applied suspension culture to hPSCs [25–27], hPSCs are usually regarded as anchorage-dependent cells onto which culture substrates are required to support for their survival and growth* in vitro*. hPSCs do not attach to normal glass, plastics, or agars, which are conventionally used for general cell culture, requiring specific ECM and other proteins, peptides, or synthetic polymers as culture substrates. Here we list the culture substrates used for maintaining hPSC self-renewal in defined culture medium in Table 1. We describe the examples and effects of each culture substrate below.

## 3. Crude Extracts Secreted by Engelbreth-Holm-Swarm (EHS) Mouse Sarcoma Cells

Since the first report of feeder-free culture conditions of hESCs [9], crude extracts from gelatinous protein mixture secreted by Engelbreth-Holm-Swarm (EHS) mouse sarcoma cells have been widely used to maintain hPSC self-renewal [28]. These extracts are commercially named as Matrigel (Corning), Cultrex BME (Trevigen), or Geltrex (Thermo Fisher Scientific), containing laminin, entactin, collagens and heparin sulfate proteoglycan, and several growth factors. Since these extracts are incompletely defined, they lead to a lot of differences and variable experimental results, making difficult to define growth factor requirements for undifferentiated growth or directed differentiation [35]. Since these extracts are not xeno-free, they must be carefully audited for animal-derived raw material controls in compliance with regulation in applying to regenerative medicine. Thus, specific ECM proteins and other synthetic culture substrates have been commonly used in recent years.

## 4. Laminin

Laminin is a heterotrimeric protein composed of *α*, *β*, and *γ*-chains. Isoforms of *α*, *β*, and *γ*-chains are found in five, four, and three genetic variants, respectively. The laminin subtypes are named according to their chain composition [53]. For example, laminin-511 consists of *α*5, *β*1, and *γ*1 chains. In the first demonstration of the feeder-free culture conditions of hESCs, the authors compared Matrigel, laminin, collagen IV, and fibronectin to hESC self-renewal [9]. They found that laminin and Matrigel support more undifferentiated hESC colonies than fibronectin or collagen IV. Subsequently, several studies examined each laminin subtype for hPSCs attachment and self-renewal. A study showed that hPSCs adhered to recombinant human laminin-332, laminin-511, and laminin-111 and these laminin isoforms were good substrates to expand undifferentiated hESCs [29]. Another study compared adhesion properties of laminin-511, laminin-332, laminin-411, laminin-111, Matrigel, or Poly-D-Lysine substrates and showed that hESCs adhered to laminin-511 significantly better than to the other substrates [30]. Several studies showed that recombinant laminin-521 successfully maintained hPSC self-renewal for long-term culture [31, 32]. Recently, recombinant E8 fragments of laminin-511 or laminin-521 (LM-E8s), which were the minimum fragments conferring integrin-binding activity, were developed [33]. These fragments adhered to hESCs and hiPSCs better than Matrigel and intact laminin isoforms and sustained long-term self-renewal of hESCs and hiPSCs in defined xeno-free media (tested in mTeSR1 (Stem Cell Technologies), StemPro (Thermo Fisher Scientific), StemFit (Ajinomoto), and serum-free medium supplemented with N2 and B27 supplemants (Thermo Fisher Scientific)) with dissociated cell passaging [34]. Although each study showed slight different results due to the preparation methods and hPSC lines, recombinant laminin-511 and laminin-521 E8 fragments seem to be the most efficient, defined culture substrates for undifferentiated hPSCs so far [53].

## 5. Other ECM Proteins

Other than laminin, several ECM proteins have been shown to support hPSC self-renewal. Vitronectin is a ECM glycoprotein of the hemopexin family and is found abundantly in serum and bone [54]. Several studies showed that vitronectin robustly supported long-term self-renewal in hPSCs for long-term culture [35–38]. Recombinant truncated vitronectin (VTN-N), which lacked N-terminal Somatomedin B domain, was designed for use with Essential 8 defined medium (Thermo Fisher Scientific or Stem Cell Technologies) and supported human pluripotent stem cell attachment and survival better than wild type vitronectin [39].

Fibronectin is a ECM glycoprotein widely expressed by multiple cell types and is critically important in vertebrate development [55]. Fibronectin also was shown to support long-term self-renewal of hPSCs in several independent studies [40–42], although some unsuccessful results in which fibronectin cannot maintain long-term hPSC self-renewal were reported [9, 35]. This discrepancy might be due to the differences of source, purification methods, or coating methods.

Type I collagen is the most abundant collagen of the human body which forms large, eosinophilic fibers known as collagen fibers [56]. Type I collagen also has been shown to support sustained self-renewal and pluripotency of hPSCs in several independent studies [20, 43]. To our knowledge, the application of other natural ECM proteins has not been reported. Some other reports developed, engineered, or modified ECM proteins to support hPSC self-renewal. A study developed a nanofibrous gelatin substrate with optimal conditions (i.e., density, solution composition, molecular weight, diameter, and crosslink time), although normal gelatin cannot support hESC attachment and growth in defined culture conditions [44]. The authors adjusted the nanofibrous gelatin with 4.6 *µ*g/cm^2^, Acetic Acid : Ethyl Acetate = 3 : 2 as the solution composition, 30 kDa as the molecular weight, 280 nm diameter, and 4 hours of crosslink to support hPSC culture. The adjusted nanofibrous gelatin substrate maintained hPSC self-renewal for a long-term culture. Another study developed a customized recombinant spider silk matrix protein, which was produced in* Escherichia coli* and can be assembled into mechanically robust films or up to meter-long fibers under nondenaturing and sterile conditions, was fused with peptide motif (i.e., PQVTRGDVFTM) from vitronectin [45]. This xeno-free recombinant protein-based substrate allowed long-term culture of undifferentiated hPSCs.

## 6. Synthetic Substrates

Other than natural or recombinant proteins, some synthetic materials have been developed to maintain hPSC self-renewal. Synthetic peptide-acrylate surfaces (PAS), which supported self-renewal of hESCs in chemically defined, xeno-free medium, were developed [46]. These surfaces are commercially sold as Synthemax (Corning), which has a synthetic surface composed of RGD (Arg-Gly-Asp) containing short peptides covalently immobilized on acrylate coating to mimic the natural cell environment. The first fully defined synthetic polymer coating, which maintained long-term growth of hESCs in different culture media, was poly[2-(methacryloyloxy)ethyl dimethyl-(3-sulfopropyl)ammonium hydroxide] (PMEDSAH) [47]. Another study developed synthetic substrates displaying heparin-binding peptides, which can interact with cell-surface glycosaminoglycans, showing that synthetic substrates that recognize cell-surface glycans can facilitate the long-term culture of pluripotent stem cells [48]. Another study developed polyvinylalcohol-co-itaconic acid hydrogels grafted with an oligopeptide derived from vitronectin (KGGPQVTRGDVFTMP) with elasticities ranging from 10.3 to 30.4 kPa storage moduli by controlling the crosslinking time [49]. The hPSCs cultured on the stiffest substrates (30.4 kPa) tended to differentiate after five days of culture, whereas the hPSCs cultured on the optimal elastic substrates (25 kPa) maintained their pluripotency for over 20 passages under xeno-free conditions. These results indicate that cell culture matrices with optimal elasticity can maintain the pluripotency of hPSCs in culture. Another study used monomers with high acrylate content, which had a moderate wettability and employed integrin *α*v*β*3 and *α*v*β*5 complexes engagement with adsorbed vitronectin, to promote colony formation [57]. The same group developed UV/ozone radiation modification of typical cell culture plastics to define a favorable surface environment for hPSC culture [50]. Another group developed a synthetic polymer interface made of hydrogel interfaces of aminopropylmethacrylamide (APMAAm) for the long-term self-renewal of human embryonic stem cells (hESCs) in defined media [51]. As the hPSCs are used in industrial and clinical applications more often, the development of defined synthetic culture substrate for hPSCs will be accelerated [47, 58]. Synthemax substrates are widely distributed; however, some of the other methods are hard to be reproduced due to the lack of availability.

## 7. E-Cadherin

E-cadherin, a Ca^2+^-dependent cell-cell adhesion molecule [59], is essential for intercellular adhesion and colony formation of hPSCs [60]. A study showed that human E-cadherin-Fc chimeric protein as a culture substrate could support the self-renewal of hESCs using completely defined culture conditions [52]. Another group showed that the combination of laminin-521 and E-cadherin Fc chimera proteins allowed clonal derivation, clonal survival, and long-term self-renewal of hPSCs under completely chemically defined conditions without ROCK inhibitors [31]. Also, the laminin-521/E-cadherin matrix allowed hESC derivation from blastocyst and single blastomere cells without a need to destroy the embryo. The use of E-cadherin proteins as culture substrates is a quite interesting method; however, the differences of gene expression, epigenetic modifications, and signaling status between normal ECM-based substrates and E-cadherin-based substrates are still unknown.

## 8. Endogenous ECM Protein Production in hPSCs

Regarding the endogenous extracellular matrix production of hPSCs, an earlier study using immunostaining methods indicated that all the ECM proteins tested in the study (i.e., firbronectin, laminin, collagen I, collagen IV, or vitronectin) were produced by only feeders or differentiated hPSCs, but not by undifferentiated hPSCs [35]. However, Miyazaki et al. showed that laminin-511 and/or laminin-521 were the most abundant laminin subtype expressed in hPSCS, using immunostaining and RT-PCR methods [29]. Another study showed that undifferentiated hESCs expressed a specific subtype of laminin-511, nidogen-1, and type IV collagen, using immunostaining and RT-PCR methods [61]. To our knowledge, the physiological roles of endogenous nidogen-1 and type IV collagen in hPSCs are still unknown. A recent study examined the production of laminin *α*5 subunit, type I collagen, fibronectin, or vitronectin in undifferentiated hPSCs and showed that only laminin *α*5 subunit was produced, using immunostaining method [62]. Taken together, undifferentiated hPSCs surely produce laminin-511 and/or laminin-521, and the production of other ECM proteins is doubtful or unexamined.

A recent study also showed that the disruption of endogenous *α*5 laminin subunit expression dramatically impaired self-renewal and increased apoptosis and that the impaired self-renewal and survival were restored by culturing on exogenous laminin-521, but not on Synthemax or vitronectin [62]. Another study also showed that the knocking down of laminin *α*5 in hESCs resulted in the reduction of* integrin α6* (*ITGA6*) and* Sox2* mRNA expression and OCT4 protein expression [63]. During differentiation, the expression pattern of laminin isoforms in hPSCs dramatically changes [64]. Together, these studies demonstrated that the endogenous laminin *α*5 expression, which forms laminin-511 or laminin-521 complexes, was specific and crucial in hPSC self-renewal and survival and that the endogenous laminin production could be substituted by the exogenous deposition of laminin-511 or laminin-521, but not by the other ECM proteins. The requirement of endogenous laminin production in hPSCs implied that the laminin could be functional to support hPSC self-renewal and survival even when other ECM proteins or other materials were used as culture substrates.

## 9. Expression Patterns and Physiological Roles of Integrin Receptors in hPSCs

Integrins are the major receptors for cell adhesion to ECM proteins, consisting of the heterodimers of the *α* and *β* subunits. Each heterodimer attaches to specific ECM proteins differently [65]. Integrin expression patterns in hPSCs have been examined in several studies. The first report of feeder-free culture conditions showed that hESCs expressed high levels of *α*6 and *β*1, moderate levels of *α*2, and low levels of *α*1, *α*3, and *β*4 integrin subunits [9]. These results suggested that the laminin-specific receptor (i.e., *α*6*β*1 integrin complex) was important for interacting hESCs with laminin. Consistent with this report, several studies, which used laminin or its fractions as culture substrates, showed that *α*6*β*1 integrin complex majorly mediates hPSC adhesion onto laminin [30, 35, 61, 66]. Another report, which used vitronectin as culture substrates, showed that *α*V*β*5 integrin, not *α*6*β*1 integrin, mediated hPSC adhesion to vitronectin [35]. Another study showed that *α*V*β*5 integrin was required for initial attachment onto vitronectin, but the inhibition of both *α*V*β*5 and *β*1 subunits was required to decrease iPSC proliferation significantly [37]. Another report, which used Matrigel as culture substrates, showed that *α*V*β*3, *α*6, *β*1, and *α*2*β*1 integrins played a significant role in the initial adhesion of the hESCs [67]. Another study showed that hiPSCs grown on the Synthemax surface primarily utilize *α*V*β*5 integrin to mediate attachment to the substrate, whereas multiple integrins were involved in cell attachment to Matrigel [68]. A recent study showed that the knockdown of integrin *α*6 in hESCs led to a reduction in NANOG, OCT4, and SOX2 levels, suggesting that integrin signaling may be crucial for maintaining hPSC self-renewal and the expression of pluripotency transcription factors [63]. Together, the laminin-specific integrin *α*6*β*1 complex must be crucial for hPSC self-renewal and survival. Other integrin complexes, such as *α*V*β*5, *α*V*β*3, and *α*2*β*1, which interact with vitronectin, fibronectin, or collagens, may be used for hPSC attachment; however, their physiological roles in hPSC self-renewal remain elusive. It will be interesting and important to examine the role of each integrin subunit in undifferentiated hPSCs by disrupting these genes.

Other than integrins, one study demonstrated that blockage of CD44, which interacts with hyaluronic acid, inhibited cell attachment in 21% O_2_ culture conditions [69]. The expression patterns and the physiological roles of other nonintegrin receptors for ECM (e.g., syndecan interacted with fibronectin and other proteins, dystroglycan interacted with laminin, or urokinase-type plasminogen activator receptor interacted with vitronectin) remain elusive.

## 10. Integrin Signaling on hPSC Self-Renewal

Molecular mechanisms mediating these culture substrates and hPSC self-renewal have been recently started to be uncovered. Generally, integrins transmit their signals via intracellular signaling proteins, such as integrin-linked kinase (ILK) and focal adhesion kinase (FAK). ILK was shown to mediate integrin signals to AKT signaling pathway, and this signaling pathway had an antagonistic effect on endoderm differentiation [70]. Other several studies showed that the activating AKT signaling promoted self-renewal and survival of hPSCs [31, 71–74]. Another study showed that soluble angiopoeitin-1 (Ang-1-) derived peptide QHREDGSQHREDGS, which interacted with *β*1 integrin, decreased hPSC apoptosis after the single-cell dissociation and that the interaction of the peptide increased ILK expression [75]. On the other hand, a recent study showed that the integrin-FAK signaling pathway was not active in undifferentiated hPSCs [63]. FAK was not phosphorylated at Y397 and was localized in the nuclei of hPSCs, interacting with OCT4 and SOX2. During differentiation, however, integrin *α*6 levels diminished, and Y397 FAK was phosphorylated. Together, these findings suggested that the integrin-ILK signaling pathway was active in order to maintain hPSC self-renewal and survival through the activation of AKT signaling pathways and that integrin-FAK signaling pathway was not active. The role of the unphosphorylated, nuclear FAK in hPSC on self-renewal should be uncovered in future.

**Figure 1:**
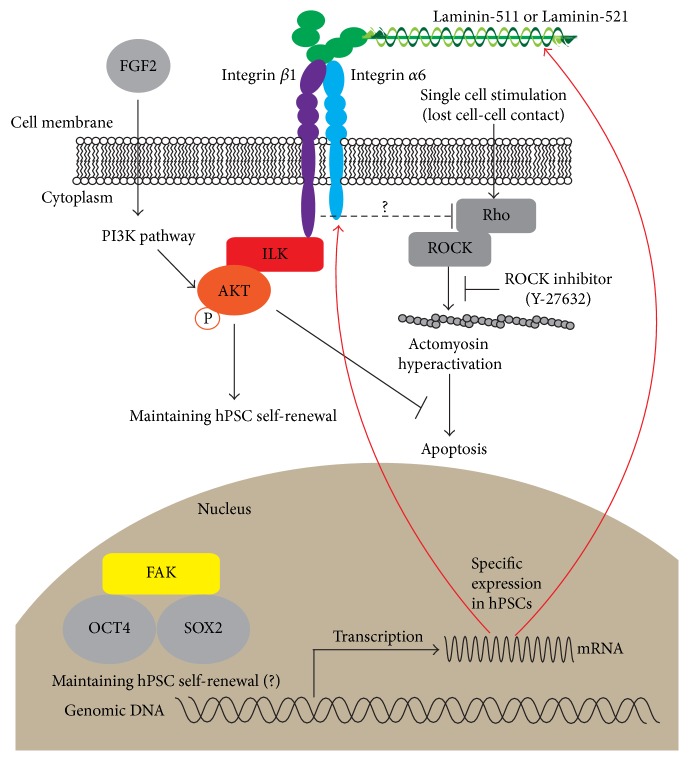
Possible schemes of ECM (laminin) integrin signaling on hPSC self-renewal.

hPSC survival from single cell is usually hard without specific chemical treatment, such as ROCK inhibitors or genetic modification [76, 77]. A recent study showed that the disruption of endogenous *α*-5 laminin production caused hPSC apoptosis and that the treatment of the laminin *α*5-deficient cells with blebbistatin or a Rho-associated kinase (ROCK) inhibitor partially restored their self-renewal and diminished apoptosis [62]. These findings implied that ECM-integrin signaling might modulate the status of the Rho-ROCK-Myosin pathway, which was considered as a major player in antiapoptosis of single-cell dissociated hPSCs [78–81]. It will be interesting to reveal the cross-talk effect of ECM-integrin signaling with these antiapoptosis pathways in hPSCs. Together, we illustrate the possible schemes of integrin signaling on self-renewal and single-cell survival of hPSCs from the recent studies described above (Figure 1).

## 11. Toward Naïve Pluripotent Stem Cells: Perspectives from Mouse Pluripotent Stem Cells

Although conventional hPSCs in bFGF-dependent culture conditions have been widely used, an alternative pluripotent state with different signal dependence has attracted much attention. The derivation of mouse epiblast stem cells (mEpiSCs) clarified that pluripotency contained two developmental stages [82, 83]. mESCs derived from preimplantation inner cell mass represent the “naïve” stage, and mEpiSCs derived from the postimplantation epiblast represent the “primed” stage. mESC self-renewal has been achieved through exposure to the leukemia inhibitory factor (LIF) [84, 85]. We also confirmed the effect of LIF on mESC self-renewal in defined culture conditions [86]. The addition of extracellular signal-regulated kinase (MEK) and glycogen synthase kinase 3 (GSK3) inhibitors (2i) in defined medium allowed the cells to attain a homogeneous ground state [87]. On the other hand, mEpiSCs are cultured in a medium containing bFGF and Activin/Nodal/TGF*β*, which is similar to hESCs culture medium. Although some early studies used LIF in feeder-free media for undifferentiated hESCs [11, 88], LIF and its downstream STAT3 signaling pathway were shown to be dispensable for maintaining primed human and primate PSC self-renewal in several independent studies [89–91]. Thus, defined hPSC culture media today usually do not contain LIF; however, reported media for naïve hPSC contain LIF, not bFGF [92–94]. STAT3 activation was reported to be crucial in reprogramming human PSCs to naïve state [95]. A recent study also showed that LIF promoted X chromosome reactivation, which was one of the characteristics of naïve pluripotency in female hPSCs [96]. These studies indicated that naïve hPSCs required different culture conditions and signaling activation status from primed hPSCs. From the similarity of the cytokine requirement and signal dependence, conventional hPSCs represent primed state, similar to mEpiSCs.

The effect of culture substrates on establishing and maintaining naïve hPSC self-renewal is still ill-defined since most of the studies still used feeder cells [92, 93]. Since the colony morphologies of naïve and primed hPSCs are considerably different (i.e., primed hPSCs form flattened colonies, whereas naïve hPSCs form dome-like 3D colonies as mESCs do), the effect of culture substrates in primed and naïve hPSCs might be different. In order to predict the respective effect of different substrates on naïve versus primed state PSCs, we introduce the findings of our previous study using mESCs in defined culture conditions [86]. We revealed that type I collagen, gelatin, or suspension were suitable to maintain mESC self-renewal and that the integrin signaling was inactivated in these conditions [97]. Conversely, laminin or fibronectin induced mEpiSC-like properties, which was featured by altered morphologies, the decreased activity of alkaline phosphatase, increased Fgf5 expression, and decreased Nanog expression. mESC expressed integrins against laminin and fibronectin, and the ECM-integrin signaling promoted differentiation into the primed state from mESCs. From the similarity between mESCs and naïve hPSCs, our findings suggest that type I collagen, gelatin, or suspension, but not laminin or fibronectin, could support naïve hPSC self-renewal and that the suppression of ECM-integrin signaling might also support the features of naïve hPSCs. It will be interesting to test the effect of different ECM proteins on human naïve pluripotency in defined culture conditions.

Examining endogenous ECM and integrin expression in naïve hPSCs and their effect on inducing and maintaining naïve pluripotency should be also interesting. As mentioned above, primed hPSCs express laminin-511 and/or laminin-521 and their receptors, integrin *α*6*β*1. However, unlike the mouse equivalent, human ICM cells lacked appreciable laminin expression [98]. Thus, there will be a possibility that naïve hPSCs might have different expression patterns of ECM proteins and integrins from primed hPSCs.

## 12. Culture Substrates Enable Controlled hPSC Differentiation

Specific culture substrates should be useful to establish efficient and robust differentiation methods. However, the effect of each material on the differentiation into specific cell types has been largely unknown yet. We introduce some of the studies to enhance the utility of specific culture substrates for inducing and maintaining specific cell types.

### 12.1. The Effect of Culture Substrates on Neural Differentiation from hPSCs

A study used defined adherent culture system to examine the effect of ECM molecules on neural differentiation of hESCs. hESC-derived differentiating embryoid bodies were plated on Poly-D-Lysine (PDL), PDL/fibronectin, PDL/laminin, type I collagen, or Matrigel in neural differentiation medium [99]. They found that neural progenitor, neuronal generation, and neurite outgrowth were significantly greater on PDL/laminin and Matrigel substrates than on other three substrates. The laminin/PDL-induced neural progenitor expansion was partially blocked by the antibody against integrin *α*6 or *β*1 subunits. Another study showed that vitronetin was expressed in the ventral part of the developing human spinal cord and profoundly promoted the derivation of oligodendrocyte progenitors that proliferated and differentiated into oligodendrocytes in response to mitogenic and survival factors [100]. These results supported the beneficial effect of vitronectin on oligodendrocytic differentiation of hESCs. Several study showed that specific synthetic polymers or peptides enhanced the proliferation and differentiation of hPSC-derived neural precursors or neurons. Polydopamine coating facilitated highly efficient, simple immobilization of neurotrophic growth factors and adhesion peptides onto polymer substrates. The growth factor or peptide-immobilized substrates greatly enhanced the differentiation and proliferation of human neural stem cells (NSCs: human fetal brain-derived NSCs and human induced pluripotent stem cell-derived NSCs) [101]. Polycaprolactone fiber matrices of different diameter (i.e., nanofibers and microfibers) and orientation (i.e., aligned and random) coated with poly-L-ornithine/laminin were developed to support the adhesion, viability, and differentiation of NSCs [102]. A synthetic polymer, poly(4-vinyl phenol) (P4VP) supported the long-term proliferation and self-renewal of hNPCs [103]. Another study showed that compliant polyacrylamide (PA) hydrogels (~0.7 kPa) functionalized with a glucosaminoglycan-binding peptide inhibited promoted highly efficient differentiation from hPSCs into postmitotic neurons even in the presence of soluble pluripotency factors [104]. Similarly, the neural induction and caudalization of hPSCs were accelerated by a synthetic microengineered substrate, consisting of soft poly-dimethylsiloxane micropost arrays (PMAs) [105].

### 12.2. The Effect of Culture Substrates on Endodermal Differentiation from hPSCs

A study examined the effect of ECM combinations and concentrations on the differentiation from several hPSC lines into definitive endoderm (DE), an early embryonic cell population fated to give rise to internal organs, such as the lung, liver, pancreas, stomach, and intestine [106]. From this screen, they identified fibronectin and vitronectin as ECM components that promoted DE differentiation. Analysis of integrin expression revealed that differentiation toward DE led to an increase in fibronectin-binding integrin *α*5 (ITGA5) and vitronectin-binding integrin *α*V (ITGAV). Another study also showed that DE highly expresses the integrins *α*V and *β*5, which have the ability to bind to vitronectin, whilst the expression of the pluripotency related laminin-binding integrin *α*3, *α*6, and *β*4 subunits was downregulated [107]. These studies indicated that ECM compatibility and integrin expression patterns changed dynamically during hPSC differentiation. During later differentiation stages toward hepatocytes, another study showed that hPSC-derived hepatoblast-like cells were maintained for more than 3 months with the ability to differentiate into both hepatocyte-like cells and cholangiocyte-like cells by culturing on the laminin-111-coated dishes [108]. On the other hand, laminin-411 and laminin-511 promoted the cholangiocyte differentiation from hepatoblast-like cells derived from hiPSCs [109]. These results showed that the difference of laminin isoforms distinguished stem cell behaviors and that specific culture substrates enabled controlling stem cell maintenance and differentiation precisely.

## 13. Application of Culture Substrates from Microfabrication to Scalable Culture Systems

Defined culture conditions with specific culture substrates should be useful to establish various scale culture technologies with specific research purposes. Indeed, a study showed that defined culture conditions enabled microfluidic perfusion culture system for hiPSCs that uses a microchamber array chip under defined ECM proteins and culture medium conditions and that fibronectin and laminin were appropriate for microfluidic devices made out of the most popular material, polydimethylsiloxane (PDMS), by screening various ECM proteins [110]. The same group also demonstrated that the differences of vitronectin and *γ*-globulin adsorption enabled patterning a PDMS surface with hPSCs [111]. Conversely, toward the development of scalable culture system of hPSCs for regenerative medicine or large-scale drug development, cost-effective, defined, and reproducible culture systems must be required. In these culture systems, the development of suitable culture substrates is critical. Compared with extracted ECM proteins, recombinant proteins and synthetic polymers have advantages in the application of these scalable culture systems.

## 14. Conclusions

As the clinical application and industrial usage of hPSCs have been advanced, numerous culture media and substrates are now being actively developed. However, some of these culture media and substrates lack the studies of the effect on cellular physiology including cellular signal status, genomic integrity, gene expression profile, or epigenetic status. So far, recombinant laminin-511 or laminin-521 proteins (or their fragments) as the culture substrates and their integrin *α*6*β*1 receptor complex seem to be most examined and to have critical roles in maintaining hPSC self-renewal. As the downstream signaling of integrin, ILK activation must be critical in maintaining hPSC self-renewal; however, the molecular mechanisms in general remain elusive. To establish reproducible and stable culture conditions to maintain hPSC self-renewal toward safe clinical application or robust drug screening from hPSCs, detailed studies on molecular mechanisms should be required to control hPSC behavior precisely. After accomplishing these studies, various culture applications from microfabrication to scalable culture of hPSC should be achieved.

## Figures and Tables

**Table 1:** Summary of culture substrates used for culturing undifferentiated hPSCs.

Name	Commercial name (if any, only major)	Material type	References
Gelatinous protein mixture	Matrigel (Corning)	Crude extract secreted by EHS mouse sarcoma cells	[9, 28]
	Geltrex (Thermo Fisher Scientific)		
	Cultrex BME (Trevigen)		

Laminin		Extracted protein	[9]

Laminin-511	LN511 (Biolamina)	Recombinant protein	[29, 30]

Laminin-521	LN521 (Biolamina)	Recombinant protein	[31, 32]
	rhLaminin-521 (Thermo Fisher Scientific)		

Laminin-E8 fragment	iMatrix-511 (Nippi)	Recombinant protein	[33, 34]

Vitronectin	Vitronectin XF (Stem Cell Technologies)	Extracted or recombinant protein	[35–38]

Truncated vitronectin	VTN-N (Thermo Fisher Scientific)	Recombinant protein	[39]

Fibronectin		Extracted protein	[40–42]

Collagen type I		Extracted protein	[20, 43]

Nanofibrous gelatin		Processed gelatin	[44]

Customized spider silk protein		Recombinant protein, containing vitronectin motif	[45]

Peptide-acrylate surfaces (PAS)	Synthemax (Corning)	Synthetic polymers with peptides	[46]

PMEDSAH		Synthetic polymer	[47]

Synthetic substrates displaying heparin-binding peptides		Synthetic polymers with peptides	[48]

Polyvinylalcohol-co-itaconic acid hydrogels grafted with aoligopeptide derived from vitronectin (KGGPQVTRGDVFTMP)		Synthetic polymers with peptides	[49]

UV/ozone radiation		Modification of typical cell culture plastics	[50]

Hydrogel interfaces of aminopropylmethacrylamide (APMAAm)		Synthetic polymers	[51]

Human E-cadherin-Fc chimeric protein		Recombinant protein	[31, 52]

## References

[1] Thomson J. A. (1998). Embryonic stem cell lines derived from human blastocysts. *Science*.

[2] Takahashi K., Tanabe K., Ohnuki M. (2007). Induction of pluripotent stem cells from adult human fibroblasts by defined factors. *Cell*.

[3] Yu J., Vodyanik M. A., Smuga-Otto K. (2007). Induced pluripotent stem cell lines derived from human somatic cells. *Science*.

[4] Mallon B. S., Hamilton R. S., Kozhich O. A. (2014). Comparison of the molecular profiles of human embryonic and induced pluripotent stem cells of isogenic origin. *Stem Cell Research*.

[5] Koyanagi-Aoi M., Ohnuki M., Takahashi K. (2013). Differentiation-defective phenotypes revealed by large-scale analyses of human pluripotent stem cells. *Proceedings of the National Academy of Sciences of the United States of America*.

[6] Riera M., Fontrodona L., Albert S. (2016). Comparative study of human embryonic stem cells (hESC) and human induced pluripotent stem cells (hiPSC) as a treatment for retinal dystrophies. *Molecular Therapy—Methods & Clinical Development*.

[7] Evans M. J., Kaufman M. H. (1981). Establishment in culture of pluripotential cells from mouse embryos. *Nature*.

[8] Martin G. R. (1981). Isolation of a pluripotent cell line from early mouse embryos cultured in medium conditioned by teratocarcinoma stem cells. *Proceedings of the National Academy of Sciences of the United States of America*.

[9] Xu C., Inokuma M. S., Denham J. (2001). Feeder-free growth of undifferentiated human embryonic stem cells. *Nature Biotechnology*.

[10] Wang G., Zhang H., Zhao Y. (2005). Noggin and bFGF cooperate to maintain the pluripotency of human embryonic stem cells in the absence of feeder layers. *Biochemical and Biophysical Research Communications*.

[11] Amit M., Shariki C., Margulets V., Itskovitz-Eldor J. (2004). Feeder layer- and serum-free culture of human embryonic stem cells. *Biology of Reproduction*.

[12] Klimanskaya I., Chung Y., Meisner L., Johnson J., West M. D., Lanza R. (2005). Human embryonic stem cells derived without feeder cells. *The Lancet*.

[13] Xu R.-H., Peck R. M., Li D. S., Feng X., Ludwig T., Thomson J. A. (2005). Basic FGF and suppression of BMP signaling sustain undifferentiated proliferation of human ES cells. *Nature Methods*.

[14] Vallier L., Alexander M., Pedersen R. A. (2005). Activin/Nodal and FGF pathways cooperate to maintain pluripotency of human embryonic stem cells. *Journal of Cell Science*.

[15] Ludwig T. E., Levenstein M. E., Jones J. M. (2006). Derivation of human embryonic stem cells in defined conditions. *Nature Biotechnology*.

[16] Lu J., Hou R., Booth C. J., Yang S., Snyder M. (2006). Defined culture conditions of human embryonic stem cells. *Proceedings of the National Academy of Sciences of the United States of America*.

[17] Liu Y., Song Z., Zhao Y. (2006). A novel chemical-defined medium with bFGF and N2B27 supplements supports undifferentiated growth in human embryonic stem cells. *Biochemical and Biophysical Research Communications*.

[18] Yao S., Chen S., Clark J. (2006). Long-term self-renewal and directed differentiation of human embryonic stem cells in chemically defined conditions. *Proceedings of the National Academy of Sciences of the United States of America*.

[19] James D., Levine A. J., Besser D., Hemmati-Brivanlou A. (2005). TGF*β*/activin/nodal signaling is necessary for the maintenance of pluripotency in human embryonic stem cells. *Development*.

[20] Furue M. K., Na J., Jackson J. P. (2008). Heparin promotes the growth of human embryonic stem cells in a defined serum-free medium. *Proceedings of the National Academy of Sciences of the United States of America*.

[21] Garcia-Gonzalo F. R., Belmonte J. C. I. (2008). Albumin-associated lipids regulate human embryonic stem cell self-renewal. *PLoS ONE*.

[22] Soh B. S., Song C. M., Vallier L. (2007). Pleiotrophin enhances clonal growth and long-term expansion of human embryonic stem cells. *Stem Cells*.

[23] Wang L., Schulz T. C., Sherrer E. S. (2007). Self-renewal of human embryonic stem cells requires insulin-like growth factor-1 receptor and ERBB2 receptor signaling. *Blood*.

[24] Nichols J., Smith A. (2009). Naive and primed pluripotent states. *Cell Stem Cell*.

[25] Amit M., Chebath J., Margulets V. (2010). Suspension culture of undifferentiated human embryonic and induced pluripotent stem cells. *Stem Cell Reviews and Reports*.

[26] Abbasalizadeh S., Larijani M. R., Samadian A., Baharvand H. (2012). Bioprocess development for mass production of size-controlled human pluripotent stem cell aggregates in stirred suspension bioreactor. *Tissue Engineering Part C: Methods*.

[27] Otsuji T. G., Bin J., Yoshimura A. (2014). A 3D sphere culture system containing functional polymers for large-scale human pluripotent stem cell production. *Stem Cell Reports*.

[28] Chatterjee P., Cheung Y., Liew C. (2011). Transfecting and nucleofecting human induced pluripotent stem cells. *Journal of Visualized Experiments*.

[29] Miyazaki T., Futaki S., Hasegawa K. (2008). Recombinant human laminin isoforms can support the undifferentiated growth of human embryonic stem cells. *Biochemical and Biophysical Research Communications*.

[30] Rodin S., Domogatskaya A., Ström S. (2010). Long-term self-renewal of human pluripotent stem cells on human recombinant laminin-511. *Nature Biotechnology*.

[31] Rodin S., Antonsson L., Niaudet C. (2014). Clonal culturing of human embryonic stem cells on laminin-521/E-cadherin matrix in defined and xeno-free environment. *Nature Communications*.

[32] Lam A. T., Li J., Chen A. K., Birch W. R., Reuveny S., Oh S. K. (2015). Improved human pluripotent stem cell attachment and spreading on xeno-free laminin-521-coated microcarriers results in efficient growth in agitated cultures. *BioResearch Open Access*.

[33] Miyazaki T., Futaki S., Suemori H. (2012). Laminin E8 fragments support efficient adhesion and expansion of dissociated human pluripotent stem cells. *Nature Communications*.

[34] Nakagawa M., Taniguchi Y., Senda S. (2014). A novel efficient feeder-free culture system for the derivation of human induced pluripotent stem cells. *Scientific Reports*.

[35] Braam S. R., Zeinstra L., Litjens S. (2008). Recombinant vitronectin is a functionally defined substrate that supports human embryonic stem cell self-renewal via *α*V*β*5 integrin. *Stem Cells*.

[36] Richards S., Leavesley D., Topping G., Upton Z. (2008). Development of defined media for the serum-free expansion of primary keratinocytes and human embryonic stem cells. *Tissue Engineering C: Methods*.

[37] Rowland T. J., Miller L. M., Blaschke A. J. (2010). Roles of integrins in human induced pluripotent stem cell growth on matrigel and vitronectin. *Stem Cells and Development*.

[38] Prowse A. B. J., Doran M. R., Cooper-White J. J. (2010). Long term culture of human embryonic stem cells on recombinant vitronectin in ascorbate free media. *Biomaterials*.

[39] Chen G., Gulbranson D. R., Hou Z. (2011). Chemically defined conditions for human iPSC derivation and culture. *Nature Methods*.

[40] Hughes C. S., Radan L., Betts D., Postovit L. M., Lajoie G. A. (2011). Proteomic analysis of extracellular matrices used in stem cell culture. *Proteomics*.

[41] Baxter M. A., Camarasa M. V., Bates N. (2009). Analysis of the distinct functions of growth factors and tissue culture substrates necessary for the long-term self-renewal of human embryonic stem cell lines. *Stem Cell Research*.

[42] Hayashi Y., Chan T., Warashina M. (2010). Reduction of *N*-glycolylneuraminic acid in human induced pluripotent stem cells generated or cultured under feeder- and serum-free defined conditions. *PLoS ONE*.

[43] Lee M., Kim Y., Ryu J. H., Kim K., Han Y.-M., Lee H. (2016). Long-term, feeder-free maintenance of human embryonic stem cells by mussel-inspired adhesive heparin and collagen type I. *Acta Biomaterialia*.

[44] Liu L., Yoshioka M., Nakajima M. (2014). Nanofibrous gelatin substrates for long-term expansion of human pluripotent stem cells. *Biomaterials*.

[45] Wu S., Johansson J., Damdimopoulou P. (2014). Spider silk for xeno-free long-term self-renewal and differentiation of human pluripotent stem cells. *Biomaterials*.

[46] Melkoumian Z., Weber J. L., Weber D. M. (2010). Synthetic peptide-acrylate surfaces for long-term self-renewal and cardiomyocyte differentiation of human embryonic stem cells. *Nature Biotechnology*.

[47] Villa-Diaz L. G., Nandivada H., Ding J. (2010). Synthetic polymer coatings for long-term growth of human embryonic stem cells. *Nature Biotechnology*.

[48] Klim J. R., Li L., Wrighton P. J., Piekarczyk M. S., Kiessling L. L. (2010). A defined glycosaminoglycan-binding substratum for human pluripotent stem cells. *Nature Methods*.

[49] Higuchi A., Kao S.-H., Ling Q.-D. (2015). Long-term xeno-free culture of human pluripotent stem cells on hydrogels with optimal elasticity. *Scientific Reports*.

[50] Saha K., Mei Y., Reisterer C. M. (2011). Surface-engineered substrates for improved human pluripotent stem cell culture under fully defined conditions. *Proceedings of the National Academy of Sciences of the United States of America*.

[51] Irwin E. F., Gupta R., Dashti D. C., Healy K. E. (2011). Engineered polymer-media interfaces for the long-term self-renewal of human embryonic stem cells. *Biomaterials*.

[52] Nagaoka M., Si-Tayeb K., Akaike T., Duncan S. A. (2010). Culture of human pluripotent stem cells using completely defined conditions on a recombinant E-cadherin substratum. *BMC Developmental Biology*.

[53] Aumailley M., Brucknertuderman L., Carter W. (2005). A simplified laminin nomenclature. *Matrix Biology*.

[54] Schvartz I., Seger D., Shaltiel S. (1999). Vitronectin. *International Journal of Biochemistry and Cell Biology*.

[55] Pankov R., Yamada K. M. (2002). Fibronectin at a glance. *Journal of Cell Science*.

[56] Di Lullo G. A., Sweeney S. M., Körkkö J., Ala-Kokko L., San Antonio J. D. (2002). Mapping the ligand-binding sites and disease-associated mutations on the most abundant protein in the human, type I collagen. *Journal of Biological Chemistry*.

[57] Mei Y., Saha K., Bogatyrev S. R. (2010). Combinatorial development of biomaterials for clonal growth of human pluripotent stem cells. *Nature Materials*.

[58] Villa-Diaz L. G., Ross A. M., Lahann J., Krebsbach P. H. (2013). Concise review: the evolution of human pluripotent stem cell culture: from feeder cells to synthetic coatings. *STEM CELLS*.

[59] Takeichi M. (1995). Morphogenetic roles of classic cadherins. *Current Opinion in Cell Biology*.

[60] Eastham A. M., Spencer H., Soncin F. (2007). Epithelial-mesenchymal transition events during human embryonic stem cell differentiation. *Cancer Research*.

[61] Evseenko D., Schenke-Layland K., Dravid G. (2009). Identification of the critical extracellular matrix proteins that promote human embryonic stem cell assembly. *Stem Cells and Development*.

[62] Laperle A., Hsiao C., Lampe M. (2015). *α*-5 Laminin synthesized by human pluripotent stem cells promotes self-renewal. *Stem Cell Reports*.

[63] Villa-Diaz L. G., Kim J. K., Laperle A., Palecek S. P., Krebsbach P. H. (2016). Inhibition of FAK signaling by integrin alpha6beta1 supports human pluripotent stem cell self-renewal. *Stem Cells*.

[64] Pook M., Teino I., Kallas A., Maimets T., Ingerpuu S., Jaks V. (2015). Changes in laminin expression pattern during early differentiation of human embryonic stem cells. *PLoS ONE*.

[65] Hynes R. O. (2002). Integrins: bidirectional, allosteric signaling machines. *Cell*.

[66] Vuoristo S., Virtanen I., Takkunen M. (2009). Laminin isoforms in human embryonic stem cells: synthesis, receptor usage and growth support. *Journal of Cellular and Molecular Medicine*.

[67] Meng Y., Eshghi S., Li Y. J., Schmidt R., Schaffer D. V., Healy K. E. (2010). Characterization of integrin engagement during defined human embryonic stem cell culture. *The FASEB Journal*.

[68] Jin S., Yao H., Weber J. L., Melkoumian Z. K., Ye K. (2012). A synthetic, xeno-free peptide surface for expansion and directed differentiation of human induced pluripotent stem cells. *PLoS ONE*.

[69] Kumar D., Gupta S., Yang Y., Forsyth N. R. (2013). *α*V*β*5 and CD44 are oxygen-regulated human embryonic stem cell attachment factors. *BioMed Research International*.

[70] Wrighton P. J., Klim J. R., Hernandez B. A., Koonce C. H., Kamp T. J., Kiessling L. L. (2014). Signals from the surface modulate differentiation of human pluripotent stem cells through glycosaminoglycans and integrins. *Proceedings of the National Academy of Sciences of the United States of America*.

[71] Li J., Wang G., Wang C. (2007). MEK/ERK signaling contributes to the maintenance of human embryonic stem cell self-renewal. *Differentiation*.

[72] Wong R. C. B., Tellis I., Jamshidi P., Pera M., Pébay A. (2007). Anti-apoptotic effect of sphingosine-1-phosphate and platelet-derived growth factor in human embryonic stem cells. *Stem Cells and Development*.

[73] Teramura T., Takehara T., Onodera Y., Nakagawa K., Hamanishi C., Fukuda K. (2012). Mechanical stimulation of cyclic tensile strain induces reduction of pluripotent related gene expressions via activation of Rho/ROCK and subsequent decreasing of AKT phosphorylation in human induced pluripotent stem cells. *Biochemical and Biophysical Research Communications*.

[74] Kinehara M., Kawamura S., Tateyama D. (2013). Protein kinase C regulates human pluripotent stem cell self-renewal. *PLoS ONE*.

[75] Dang L. T. H., Feric N. T., Laschinger C. (2014). Inhibition of apoptosis in human induced pluripotent stem cells during expansion in a defined culture using angiopoietin-1 derived peptide QHREDGS. *Biomaterials*.

[76] Watanabe K., Ueno M., Kamiya D. (2007). A ROCK inhibitor permits survival of dissociated human embryonic stem cells. *Nature Biotechnology*.

[77] Chen D., Sun Y., Deng C. X., Fu J. (2015). Improving survival of disassociated human embryonic stem cells by mechanical stimulation using acoustic tweezing cytometry. *Biophysical Journal*.

[78] Pakzad M., Totonchi M., Taei A., Seifinejad A., Hassani S. N., Baharvand H. (2010). Presence of a ROCK inhibitor in extracellular matrix supports more undifferentiated growth of feeder-free human embryonic and induced pluripotent stem cells upon passaging. *Stem Cell Reviews and Reports*.

[79] Ohgushi M., Matsumura M., Eiraku M. (2010). Molecular pathway and cell state responsible for dissociation-induced apoptosis in human pluripotent stem cells. *Cell Stem Cell*.

[80] Harb N., Archer T. K., Sato N. (2008). The Rho-Rock-Myosin signaling axis determines cell-cell integrity of self-renewing pluripotent stem cells. *PLoS ONE*.

[81] Chen G., Hou Z., Gulbranson D. R., Thomson J. A. (2010). Actin-myosin contractility is responsible for the reduced viability of dissociated human embryonic stem cells. *Cell Stem Cell*.

[82] Brons I. G. M., Smithers L. E., Trotter M. W. B. (2007). Derivation of pluripotent epiblast stem cells from mammalian embryos. *Nature*.

[83] Tesar P. J., Chenoweth J. G., Brook F. A. (2007). New cell lines from mouse epiblast share defining features with human embryonic stem cells. *Nature*.

[84] Smith A. G., Heath J. K., Donaldson D. D. (1988). Inhibition of pluripotential embryonic stem cell differentiation by purified polypeptides. *Nature*.

[85] Williams R. L., Hilton D. J., Pease S. (1988). Myeloid leukaemia inhibitory factor maintains the developmental potential of embryonic stem cells. *Nature*.

[86] Furue M., Okamoto T., Hayashi Y. (2005). Leukemia inhibitory factor as an anti-apoptotic mitogen for pluripotent mouse embryonic stem cells in a serum-free medium without feeder cells. *In Vitro Cellular and Developmental Biology—Animal*.

[87] Ying Q.-L., Wray J., Nichols J. (2008). The ground state of embryonic stem cell self-renewal. *Nature*.

[88] Li Y., Powell S., Brunette E., Lebkowski J., Mandalam R. (2005). Expansion of human embryonic stem cells in defined serum-free medium devoid of animal-derived products. *Biotechnology and Bioengineering*.

[89] Sumi T., Fujimoto Y., Nakatsuji N., Suemori H. (2004). STAT3 is dispensable for maintenance of self-renewal in nonhuman primate embryonic stem cells. *Stem Cells*.

[90] Dahéron L., Opitz S. L., Zaehres H. (2004). LIF/STAT3 signaling fails to maintain self-renewal of human embryonic stem cells. *Stem Cells*.

[91] Humphrey R. K., Beattie G. M., Lopez A. D. (2004). Maintenance of pluripotency in human embryonic stem cells is STAT3 independent. *Stem Cells*.

[92] Hanna J., Cheng A. W., Saha K. (2010). Human embryonic stem cells with biological and epigenetic characteristics similar to those of mouse ESCs. *Proceedings of the National Academy of Sciences of the United States of America*.

[93] Buecker C., Chen H.-H., Polo J. M. (2010). A murine ESC-like state facilitates transgenesis and homologous recombination in human pluripotent stem cells. *Cell Stem Cell*.

[94] Hu Z., Pu J., Jiang H. (2015). Generation of naivetropic induced pluripotent stem cells from Parkinson's disease patients for high-efficiency genetic manipulation and disease modeling. *Stem Cells and Development*.

[95] Chen H., Aksoy I., Gonnot F. (2015). Reinforcement of STAT3 activity reprogrammes human embryonic stem cells to naive-like pluripotency. *Nature Communications*.

[96] Tomoda K., Takahashi K., Leung K. (2012). Derivation conditions impact x-inactivation status in female human induced pluripotent stem cells. *Cell Stem Cell*.

[97] Hayashi Y., Furue M. K., Okamoto T. (2007). Integrins regulate mouse embryonic stem cell self-renewal. *STEM CELLS*.

[98] Niakan K. K., Eggan K. (2013). Analysis of human embryos from zygote to blastocyst reveals distinct gene expression patterns relative to the mouse. *Developmental Biology*.

[99] Ma W., Tavakoli T., Derby E., Serebryakova Y., Rao M. S., Mattson M. P. (2008). Cell-extracellular matrix interactions regulate neural differentiation of human embryonic stem cells. *BMC Developmental Biology*.

[100] Gil J.-E., Woo D.-H., Shim J.-H. (2009). Vitronectin promotes oligodendrocyte differentiation during neurogenesis of human embryonic stem cells. *FEBS Letters*.

[101] Yang K., Lee J. S., Kim J. (2012). Polydopamine-mediated surface modification of scaffold materials for human neural stem cell engineering. *Biomaterials*.

[102] Mahairaki V., Lim S. H., Christopherson G. T. (2011). Nanofiber matrices promote the neuronal differentiation of human embryonic stem cell-derived neural precursors *in vitro*. *Tissue Engineering Part A*.

[103] Tsai Y., Cutts J., Kimura A., Varun D., Brafman D. A. (2015). A chemically defined substrate for the expansion and neuronal differentiation of human pluripotent stem cell-derived neural progenitor cells. *Stem Cell Research*.

[104] Musah S., Wrighton P. J., Zaltsman Y. (2014). Substratum-induced differentiation of human pluripotent stem cells reveals the coactivator YAP is a potent regulator of neuronal specification. *Proceedings of the National Academy of Sciences of the United States of America*.

[105] Sun Y., Yong K. M. A., Villa-Diaz L. G. (2014). Hippo/YAP-mediated rigidity-dependent motor neuron differentiation of human pluripotent stem cells. *Nature Materials*.

[106] Brafman D. A., Phung C., Kumar N., Willert K. (2013). Regulation of endodermal differentiation of human embryonic stem cells through integrin-ECM interactions. *Cell Death and Differentiation*.

[107] Wong J. C. Y., Gao S. Y., Lees J. G., Best M. B., Wang R., Tuch B. E. (2010). Definitive endoderm derived from human embryonic stem cells highly express the integrin receptors *α*V and *β*5. *Cell Adhesion and Migration*.

[108] Takayama K., Nagamoto Y., Mimura N. (2013). Long-term self-renewal of human ES/iPS-derived hepatoblast-like cells on human laminin 111-coated dishes. *Stem Cell Reports*.

[109] Takayama K., Mitani S., Nagamoto Y. (2016). Laminin 411 and 511 promote the cholangiocyte differentiation of human induced pluripotent stem cells. *Biochemical and Biophysical Research Communications*.

[110] Yoshimitsu R., Hattori K., Sugiura S. (2014). Microfluidic perfusion culture of human induced pluripotent stem cells under fully defined culture conditions. *Biotechnology and Bioengineering*.

[111] Yamada R., Hattori K., Tachikawa S. (2014). Control of adhesion of human induced pluripotent stem cells to plasma-patterned polydimethylsiloxane coated with vitronectin and *γ*-globulin. *Journal of Bioscience and Bioengineering*.

